# A Rare Case of Epstein-Barr Virus-Encoded RNA In Situ Hybridization-Negative Lymphoepithelial Carcinoma With a Nonneoplastic Scar: A Case Report and Literature Review

**DOI:** 10.7759/cureus.90617

**Published:** 2025-08-20

**Authors:** Yusuke Nabe, Hiroshi Mizuuchi, Masaaki Inoue, Junichi Yoshida

**Affiliations:** 1 Department of Chest Surgery, Shimonoseki City Hospital, Yamaguchi, JPN

**Keywords:** ebv-encoded small rna, epstein-barr virus, lung cancer, nodule, nonneoplastic scarring, pulmonary lymphoepithelial carcinoma, tumor

## Abstract

The etiology of pulmonary lymphoepithelioma-like carcinoma (PLELC) remains unclear. We report the case of an 83-year-old man with a history of smoking who presented without any significant complaints. Computed tomography (CT) performed to investigate emphysema revealed a nodular shadow in the left upper lobe. A subsequent CT performed three months later showed an increase in the size of the nodule, raising suspicion of lung cancer. Thoracoscopic partial left upper lobectomy was conducted, and pathological examination confirmed left PLELC with a granulomatous reaction, classified as pT1bN0M0, stage IA2. Despite negative Epstein-Barr virus (EBV)-encoded small RNA (EBER) in situ hybridization, serum anti-viral capsid antigen immunoglobulin G (VCA IgG) and anti-Epstein-Barr nuclear antigen (EBNA) IgG antibodies were positive, indicating a history of Epstein-Barr virus infection. The postoperative course was uneventful, and the patient was discharged on the sixth postoperative day (POD). No adjuvant chemotherapy was administered, and the patient remained disease-free nine months after surgery. The interplay between scarring and carcinogenesis in PLELC remains poorly understood, highlighting the need for further case investigations.

## Introduction

Lymphoepithelial-like carcinoma was first reported by Bégin et al. in 1987 [1] and was subsequently designated pulmonary lymphoepithelioma-like carcinoma (PLELC) [2]. According to the revised World Health Organization (WHO) pathological classification, lymphoepithelial carcinoma is identified as a subtype of squamous cell carcinoma (SCC) [3]. PLELC constitutes <1% of all malignant lung tumors [4]. The tumor size at initial diagnosis varies considerably, ranging from 7 mm to 110 mm [5]. The majority of cases are observed in Asian nonsmokers [6]. Histologically, PLELC is characterized by undifferentiated carcinoma with prominent lymphocytic infiltration, with morphological and molecular characteristics resembling those of nasopharyngeal carcinoma [4].

Approximately 95%-100% of PLELC cases in Asian patients are associated with Epstein-Barr virus (EBV); however, the association between EBV infection and PLELC is not generally recognized in Caucasian patients. Consequently, a positive EBV-encoded small RNA (EBER) in situ hybridization test is considered desirable but not essential according to the WHO diagnostic criteria [7]. PLELC exhibits a strong pathogenic association with EBV infection, particularly in Asian populations, suggesting a distinct oncogenic pathway triggered by viral oncoproteins, such as latent membrane protein 1 (LMP1) and EBERs [8].

EBV can infect lung epithelial cells either latently or lytically, thereby inhibiting innate and adaptive immunity [9]. However, PLELC may involve oncogenic factors other than EBV infection [10]. Nevertheless, the etiology of PLELC remains unclear.

Herein, we report a rare case of EBER in situ hybridization-negative lymphoepithelial carcinoma with nonneoplastic scarring, discuss its characteristics, and review the literature.

## Case presentation

**Figure 1:**
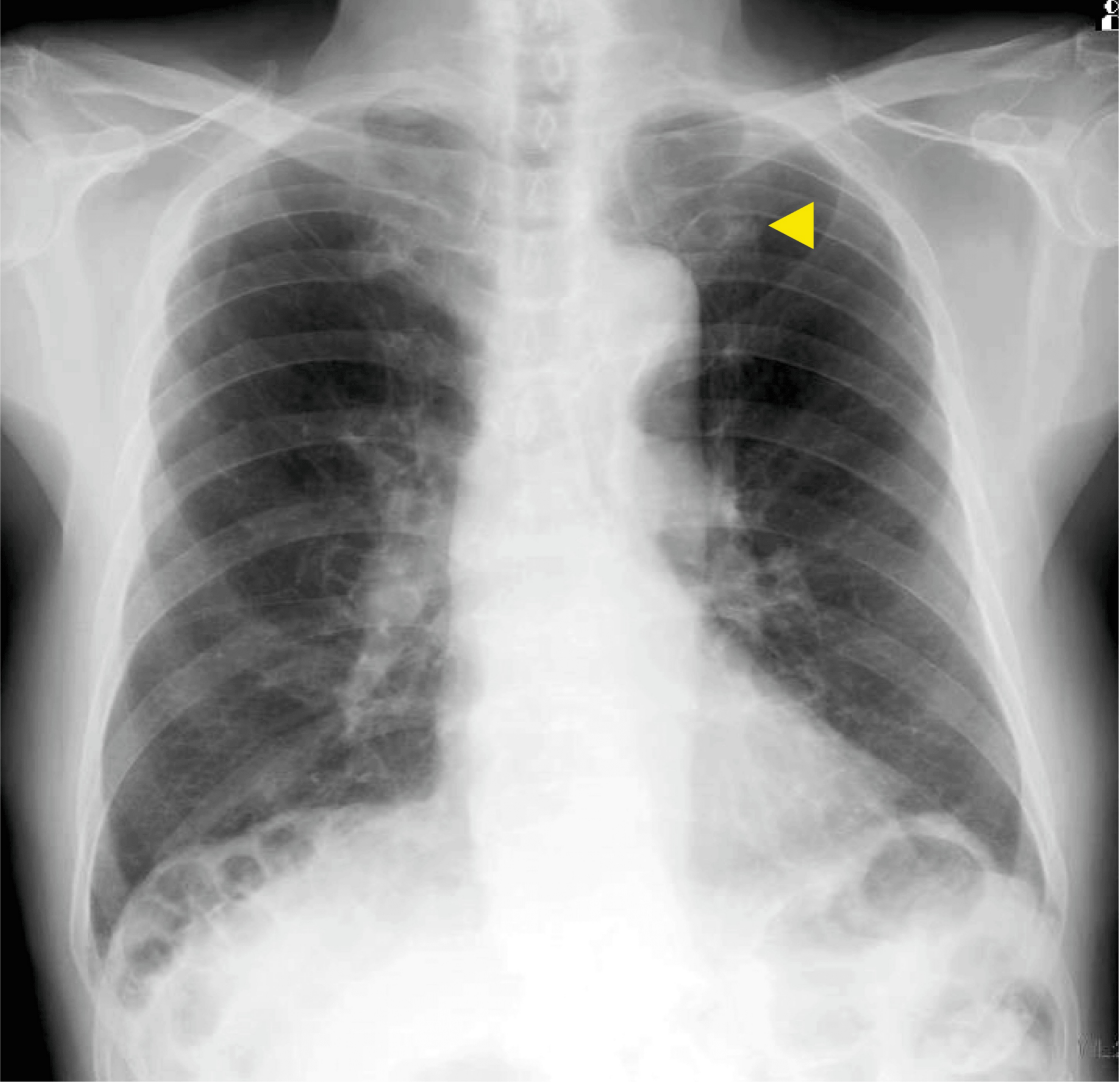
Initial chest radiography findings A 12 mm × 10 mm nodular shadow was observed in the upper left lung lobe (▶).

**Figure 2:**
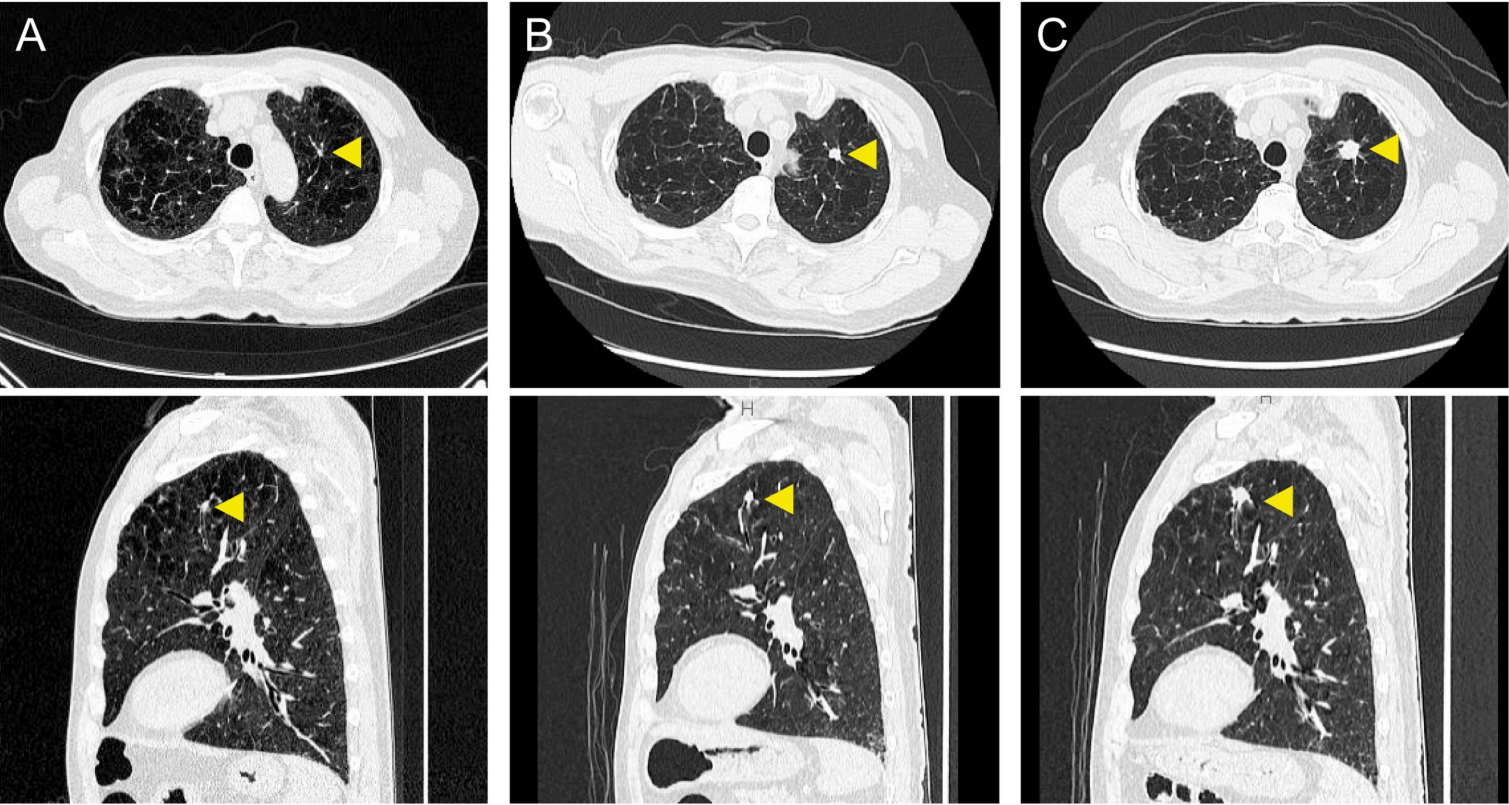
Comparison of CT findings from initial and subsequent examinations A: A chest CT scan performed two years and two months earlier detected a 4 mm × 3 mm nodule (▶). B: A chest CT scan conducted three months earlier revealed a 7 mm × 6 mm nodule (▶). C: A chest CT scan at the latest visit showed a 15 mm × 11 mm nodule in the left upper lobe (▶). CT: computed tomography

The patient was an 83-year-old man with no major complaints. A computed tomography (CT) scan conducted at the Respiratory Medicine Department of our hospital to assess emphysema revealed a nodular shadow in the left upper lobe. A subsequent CT scan performed three months later indicated an increased size of the nodular shadow, raising suspicion of lung cancer. Therefore, the patient was referred to our department. The patient had a history of emphysema, bronchial asthma, ossification of the posterior longitudinal ligament of the neck, and lumbar spinal canal stenosis. The patient had a smoking history of 20 cigarettes per day from the age of 20 years up to the age of 80. The tumor marker level was slightly elevated, with a SCC antigen level of 3.2 ng/mL (normal range: 0.6-2.3 ng/mL). Chest radiography identified a 12 mm × 10 mm nodular shadow in the upper left lung lobe (Figure 1). A chest CT scan revealed a 15 mm × 11 mm nodule in the upper left lobe, which showed an increased size compared with the findings of the CT scan performed three months previously. A CT scan conducted two years and two months earlier for fever symptoms had revealed a 4 mm × 3 mm nodule, but no indication of cancer was reported at that time (Figure 2). Blood examination at the time showed no increased white blood cell count, and tests for coronavirus and influenza virus were negative. The preoperative diagnosis was left upper lobe lung cancer (cT1bN0M0, stage IA2). No abnormalities in cardiac or pulmonary function were noted. However, the patient had ossification of the posterior longitudinal ligament in the neck and lumbar spinal canal stenosis, necessitating wheelchair use.

**Figure 3:**
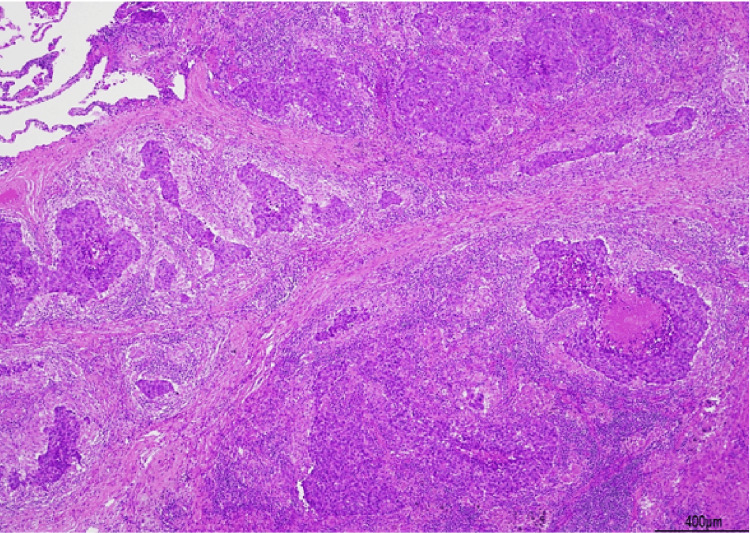
Tumor and nonneoplastic scar findings A combination of tumor tissue and nonneoplastic scars was observed.

**Figure 4:**
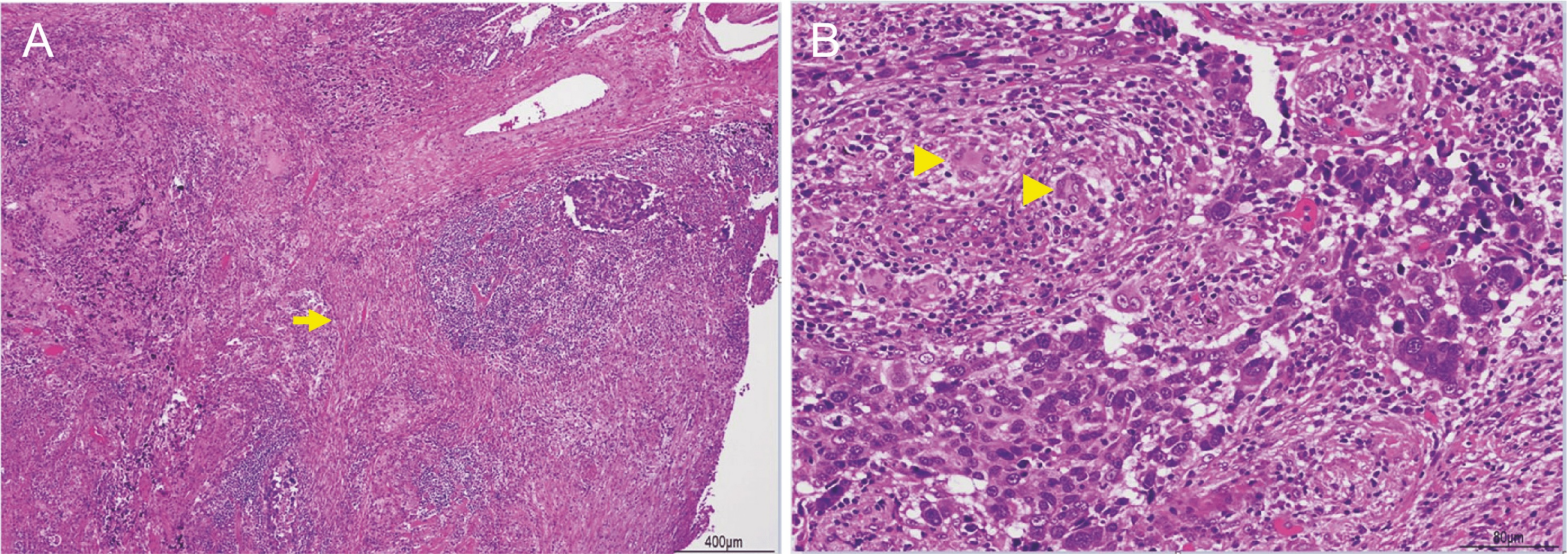
Pathological findings via hematoxylin and eosin staining A: The tumor was characterized by solid alveolar proliferation of large, atypical cells with basophilic cytoplasm, round and slightly vacuolated nuclei, and relatively clear nucleoli, appearing syncytial under low magnification. B: Prominent lymphocytic infiltration and epithelioid granulomatous reaction were present around the tumor. ➡: nonneoplastic scars, ▶: granulomatous reaction

Considering the limited mobility of this patient, reductive surgery and thoracoscopic partial resection of the left upper lobe of the lung were performed. The surgery lasted 59 minutes, with a blood loss volume of 1 mL. As the procedure was planned to conclude with partial resection, a rapid intraoperative pathological diagnosis was not conducted. The postoperative pathological diagnosis was lymphoepithelial carcinoma of the left lung with granulomatous reaction (pT1bN0M0, stage IA2). The overall dimensions were 12 mm × 7 mm × 7 mm, with no evidence of pleural invasion, intrapulmonary metastasis, or vascular invasion. The tumor exhibited solid alveolar proliferation of large, atypical cells characterized by basophilic cytoplasm, round, slightly vacuolated nuclei, relatively clear nucleoli, and a syncytial appearance at low magnification. Notably, the tumor appeared to have developed from a nonneoplastic scar (Figure 3). Prominent lymphocytic infiltration and epithelioid granulomatous reactions in the surrounding area are illustrated in Figure 4.

**Figure 5:**
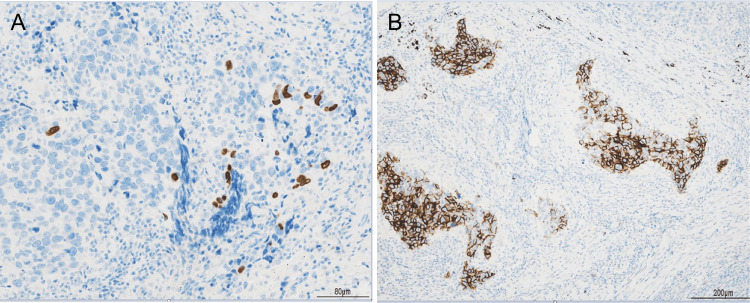
Immunostaining results A: p40 positivity was observed in a small percentage of cells. B: CD56 positivity was noted in some cell clusters.

**Figure 6:**
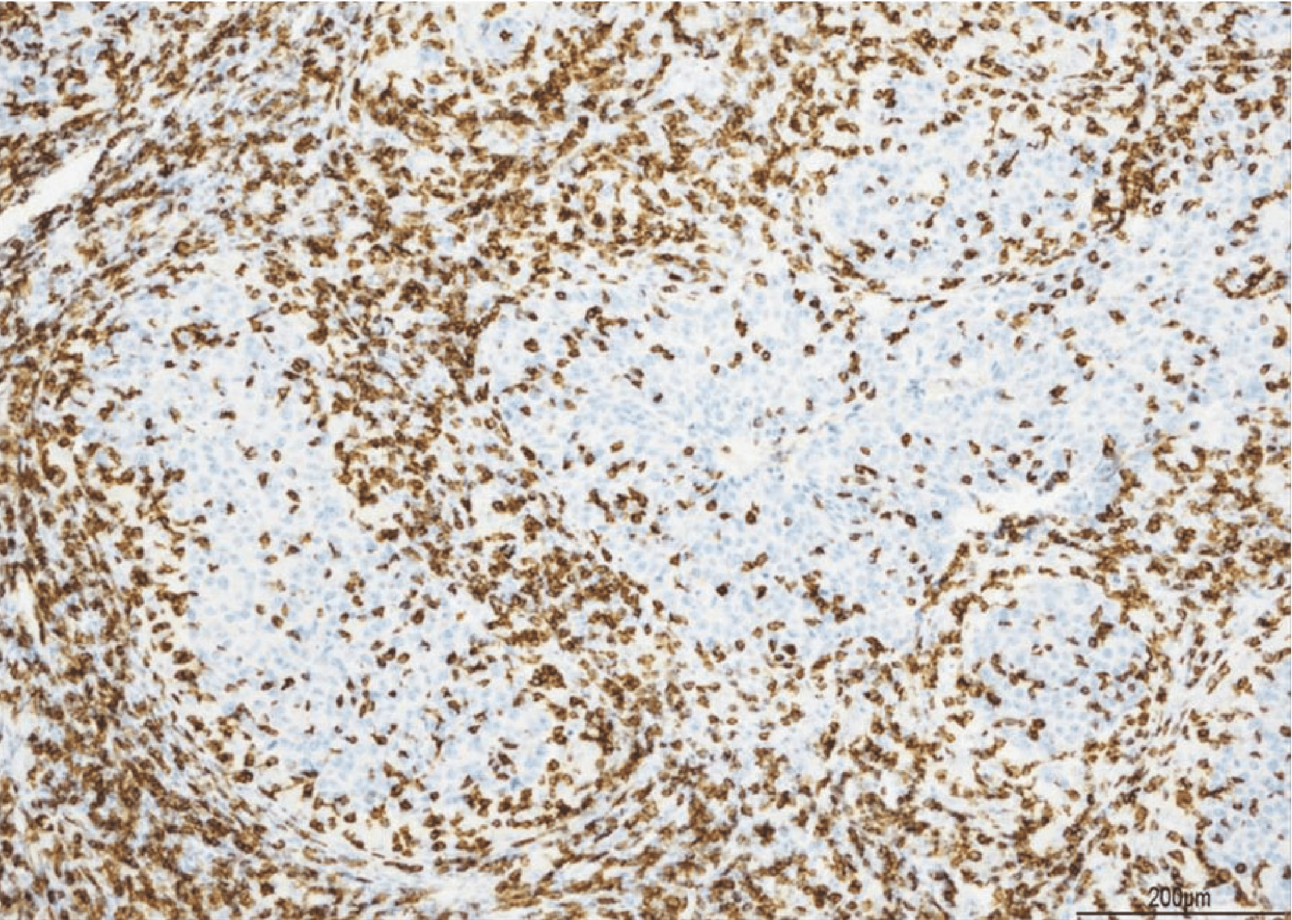
CD8 staining of infiltrating lymphocytes The majority of infiltrating lymphocytes were CD8 positive.

**Figure 7:**
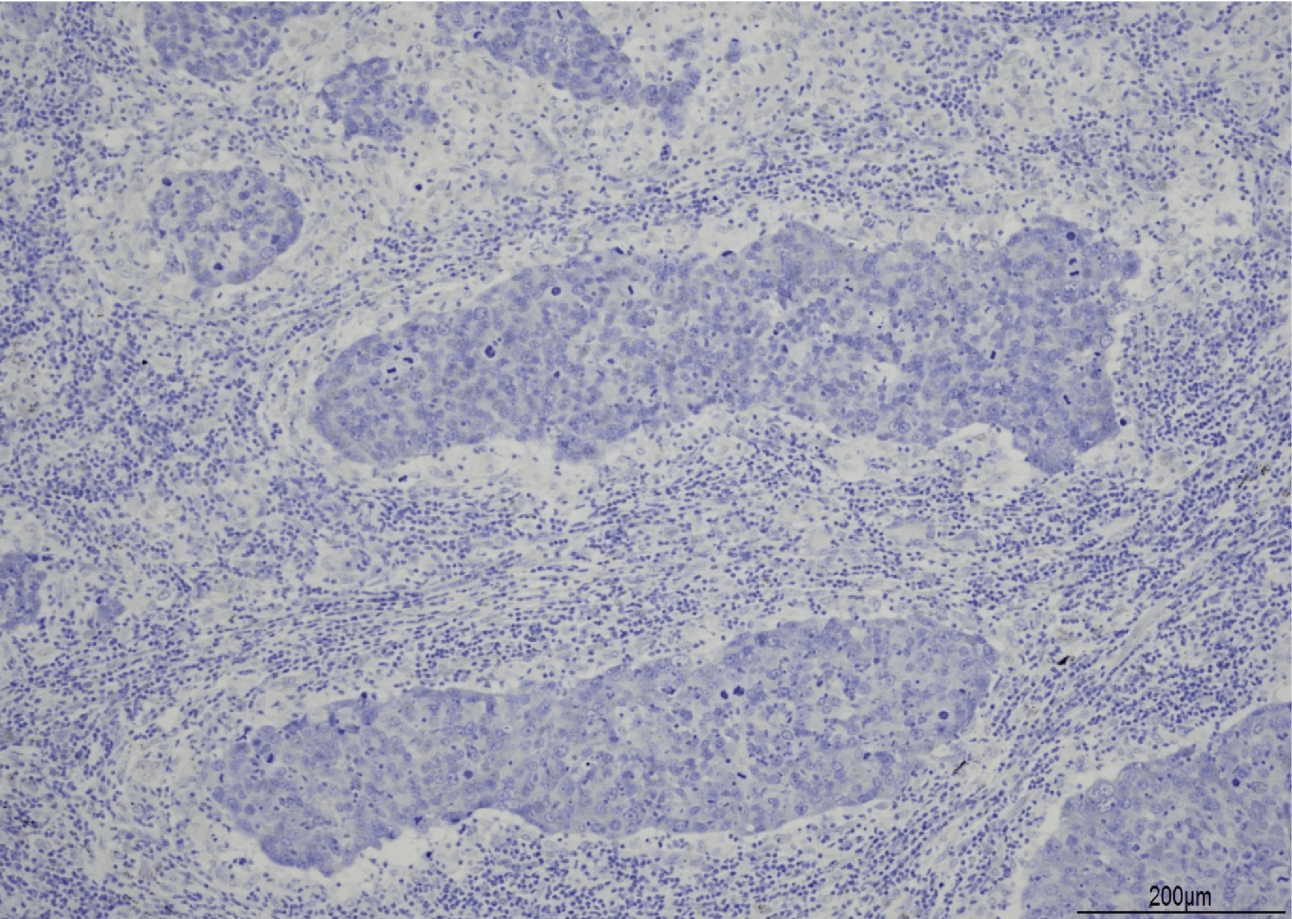
EBER in situ hybridization results EBER in situ hybridization test yielded negative results. EBER: Epstein-Barr virus-encoded small RNA

Immunostaining revealed p40 positivity in a small percentage of cells, with CD56 positivity being detected in some clusters (Figure 5). Thyroid transcription factor-1, chromogranin A, and synaptophysin were negative. The majority of infiltrating lymphocytes were CD8 positive (Figure 6). The programmed death-ligand 1 (PD-L1) tumor proportion score indicated that ≥75% of the cells were positive, whereas the AmoyDX test was negative. EBER in situ hybridization yielded negative results (Figure 7); however, serum anti-viral capsid antigen immunoglobulin G (VCA IgG) and anti-Epstein-Barr nuclear antigen (EBNA) IgG antibodies were positive, indicating prior EBV infection. The patient experienced no postoperative complications. The left chest drain was removed on postoperative day (POD) 1, and the patient was discharged on POD 6. Given the early stage of lung cancer, no adjuvant chemotherapy was administered, and the patient was monitored as an outpatient for potential recurrence. Nine months after surgery, the patient exhibited no evidence of disease recurrence.

## Discussion

Latent EBV infection during childhood in patients who develop PLELC may be associated with an underlying genetic predisposition, with EBV oncoproteins and their downstream signaling pathways subsequently promoting the transformation of airway epithelium to malignant epithelial carcinoma [9]. In the present case, EBER in situ hybridization was negative, and EBV infection in the tumor was not observed. Nevertheless, the presence of serum anti-VCA IgG and anti-EBNA IgG antibodies, along with a history of EBV infection, was noted. Additionally, active lymphoplasmacytic stromal infiltration associated with syncytial formation of the tumor aided in excluding nuclear protein in testis carcinoma [11]. Because PLELC is a subtype of squamous cell carcinoma, by definition, p40 expression should be diffuse. However, in this case, p40 positivity was only observed in a small percentage of cells and was not diffuse. Large cell carcinoma and high-grade neuroendocrine tumors were considered as possible candidates for differential diagnosis, and these needed to be excluded. We incorporated immunostaining for thyroid transcription factor-1, chromogranin A, and synaptophysin, which yielded negative results, consequently excluding the possibility of high-grade neuroendocrine tumors. Although additional immunostaining for pancytokeratin and squamous cell markers, such as CK5/6 and p63, could have potentially improved diagnostic accuracy, these tests were not conducted in this case. Notably, marked PD-L1 positivity, accompanied by infiltrating CD8-positive lymphocytes, was observed, consistent with previous reports on PLELC [11-13]. While PLELC tumor cells have been reported to express high PD-L1 levels, tumor-specific CD8+ tumor-infiltrating lymphocytes (TILs) only partially express PD-1, resulting in a limited response to immune checkpoint inhibitors [13]. Although 95% of Japanese adults are infected with EBV, the EBV antibody positivity rate among 17- to 18-year-old Americans is 26%-38% [14]. Several cases of lymphoepithelioma-like carcinoma (LELC) have been reported in Caucasian patients, some of which were EBER in situ hybridization-positive, whereas others were negative [11,15,16]. Consequently, EBV infection does not solely account for the etiology of LELC, suggesting the involvement of additional factors beyond EBV infection.

In the present case, a CT scan performed two years and two months earlier, prompted by fever evaluation, revealed a nodule suggestive of scarring. Owing to the absence of testing, it remains unclear whether the fever was related to EBV infection. We proposed two hypotheses: (1) PLELC was caused by EBV infection, but the EBER protein was no longer detectable following malignant transformation; and (2) PLELC was triggered by the scar tissue surrounding the tumor. As previously highlighted, various documented cases exist in Caucasian patients in which an EBV infection link has not been established; however, none of these reports conducted detailed investigations into the travel history of patients to Asia or prior EBV infection. If EBER in situ hybridization results are negative, a history of travel to Asia or EBV infection should be considered. Despite previous studies on scar tissue-related lung cancer [17,18], no reports have explicitly delineated the oncogenic process. Demonstrating these two hypotheses in the present study proved challenging. Hence, including more cases in future studies is crucial for elucidating the underlying mechanisms of these hypotheses.

## Conclusions

We report an interesting case in which a nodule, initially not suspected to be lung cancer on CT examination, exhibited growth approximately two years later and was diagnosed as lymphoepithelial carcinoma adjacent to a nonneoplastic scar morphological feature. Primary PLELC with scarring is extremely rare, with scar-associated PLELC representing a possible pathogenic mechanism. Consequently, further cases should focus on this point.
